# Association between the microbiomes of tonsil and saliva samples isolated from pediatric patients subjected to tonsillectomy for the treatment of tonsillar hyperplasia

**DOI:** 10.1038/s12276-020-00487-6

**Published:** 2020-09-04

**Authors:** Da Hyeon Choi, Jiwon Park, Ju Kwang Choi, Kyeong Eun Lee, Won Hee Lee, Jinho Yang, Ju Yeon Lee, Yoon Jeong Park, Chan Oh, Ho-Ryun Won, Bon Seok Koo, Jae Won Chang, Yoon Shin Park

**Affiliations:** 1 Department of Microbiology, School of Biological Sciences, College of Natural Sciences, Chungbuk National University, Cheongju, 28644 Republic of Korea; 2 Institute of MD Healthcare Inc, Seoul, 03923 Republic of Korea; 3 Central Research Institute, Nano Intelligent Biomedical Engineering Corporation (NIBEC), School of Dentistry, Seoul National University, Seoul, 03080 Republic of Korea; 4 Department of Dental Regenerative Bioengineering and Dental Research Institute, School of Dentistry, Seoul National University, Seoul, 03080 Republic of Korea; 5 Department of Otolaryngology-Head and Neck Surgery, Chungnam National University College of Medicine, Daejeon, 35015 Republic of Korea

**Keywords:** Comparative genomics, Metagenomics

## Abstract

Oral microbes have the capacity to spread throughout the gastrointestinal system and are strongly associated with multiple diseases. Given that tonsils are located between the oral cavity and the laryngopharynx at the gateway of the alimentary and respiratory tracts, tonsillar tissue may also be affected by microbiota from both the oral cavity (saliva) and the alimentary tract. Here, we analyzed the distribution and association of the microbial communities in the saliva and tonsils of Korean children subjected to tonsillectomy because of tonsil hyperplasia (*n* = 29). The microbiome profiles of saliva and tonsils were established via 16S rRNA gene sequencing. Based on the alpha diversity indices, the microbial communities of the two groups showed high similarities. According to Spearman’s ranking correlation analysis, the distribution of *Treponema*, the causative bacterium of periodontitis, in saliva and tonsils was found to have a significant positive correlation. Two representative microbes, *Prevotella* in saliva and *Alloprevotella* in tonsils, were negatively correlated, while *Treponema 2* showed a strong positive correlation between saliva and tonsils. Taken together, strong similarities in the microbial communities of the tonsils and saliva are evident in terms of diversity and composition. The saliva microbiome is expected to significantly affect the tonsil microbiome. Furthermore, we suggest that our study creates an opportunity for tonsillar microbiome research to facilitate the development of novel microbiome-based therapeutic strategies.

## Oral microbes: The populations of tonsils and saliva

Analysing the microbes on tonsils and in saliva from the mouths of children in South Korea provides comparisons of the microbial populations in these distinct but nearby regions, offering possible guidance for developing new therapies for various oral diseases. Researchers in South Korea led by Yoon Shin Park at Chungbuk National University, Cheongju, and Jae Won Chang at Chungnam National University, Daejeon, examined surgically removed tonsils from 29 patients, together with samples of the patients’ saliva. They identified many specific microbe types, finding broad similarities between the populations of an individual’s tonsils and saliva. They also identified positive and negative correlations between the presence of specific microbes in the two populations. The results could guide future treatment choices, including the use of microbes to treat disease.

## Introduction

Human tonsils are lymphoid epithelial tissues of the oral mucosa around the oropharynx and nasopharynx that protect the body from pathogen invasion through the mouth and nose. Tonsils are a component of the immune system that develops from 2 years of age, grow until puberty and gradually undergo atrophy thereafter. More frequently in children than in adults, the tonsils can become abnormally enlarged (adenotonsillar hyperplasia) or inflamed (tonsillitis), and these conditions can be surgically treated by the removal of the tonsils (tonsillectomy). Abnormally enlarged tonsillar tissue of pediatric patients is generally caused by hypertrophy rather than inflammation. Hyperplasia of the tonsils is a cause of obstructive sleep apnea and recurrent infection of the upper airways. The most common pediatric operative procedure for tonsillar hyperplasia is tonsillectomy^1^.

Oral bacteria travel throughout the body and are significantly associated with human diseases. Approximately 8 × 10^11^ bacterial cells per day flow from the mouth to the alimentary tract, and several studies have reported associations of microbes between these regions^2–4^. Although orally ingested *Porphyromonas gingivalis* affects intestinal microflora and a variety of oral microbes reach the gut microbiota, only a subset of these bacteria colonize the gut under dysbiotic conditions^5^. Microbiome analysis at 18 body sites of 200 individuals showed that the core operational taxonomic units (OTUs), shared by more than 95% of subjects, were more abundant in the oral cavity than the rest of the body^6^.

Significant quantities of pathogenic bacteria are present in hypertrophic tonsils and adenoids compared to their normal controls^7–11^. An investigation of the tonsillar surface and core of children with recurrent tonsillitis and tonsillar hypertrophy identified *Haemophilus influenzae* and *Bacteroides melaninogenicus* as the most prevalent bacteria^12^. Jeong et al.^13^ examined the differences in bacterial pathogen constituents of recurrent tonsillitis and tonsillar hypertrophy using the cultivation method in relation to age, season, and antibiotic sensitivity in patients subjected to tonsillectomy. *H. influenzae*, *Streptococcus pyogenes*, *Staphylococcus aureus*, and *Streptococcus pneumoniae* were isolated as representative abundant microbes in tonsils.

Until recently, microbiomes have been analyzed using cultivation and PCR methods. Using the cultivation method, a microbiological study on the core of tonsils was performed in 50 children with recurrent tonsillitis^14^. The predominantly isolated microbes were *S. aureus*, *Moraxella catarrhalis*, *Peptostreptococcus*, pigmented *Prevotella* and *Porphyromonas*, and *Fusobacterium* species. However, culture-based studies present a potentially biased assessment of microbial diversity due to the targeting of limited bacterial groups. More recently, 16S rRNA gene-based pyrosequencing and culture-independent techniques have been applied to survey bacterial communities.

Given that tonsils are located between the oral cavity and the laryngopharynx at the gateway of the alimentary and respiratory tracts, the tonsil lymphoid system with antigen capture M cells similar to those of Peyer’s patches in the gut is among the first handling sites for microbial agents and antigens in the human body. The microbial community of saliva can, therefore, affect that of tonsils and vice versa. To date, oral microbiome studies have mainly targeted saliva samples. Moreover, the majority of tonsil microbiota studies have focused on acute inflammatory disease and not normal conditions. Since deeply branched crypts of the tonsillar epithelium provide optimal spaces for microbiota colonization, it is speculated that the tonsil microbiome has distinct properties from other oral microbiomes. However, no studies have examined this theory, and further investigation of the correlation between the microbiomes of saliva and tonsils is warranted.

Here, we focused on the distribution and correlations of microbiota in the saliva and tonsillar tissues of young tonsillectomy patients based on the evaluation of the V3–V5 region of 16S rRNA genes to examine the hypothesis that the microbiome is associated with tonsillar hyperplasia in children. To our knowledge, this is the first comparative analysis of microbiome portraits in the tonsils and saliva of children subjected to tonsillectomy in South Korea. The elucidation of the microbiome constituents of saliva and tonsils in pediatric cases with tonsillar hypertrophy should aid in the treatment of oral disease and the development of therapeutic agents.

## Materials and methods

### Sample collection

Among the 45 participants who underwent a tonsillectomy at Chungnam National University Hospital (CNUH, Daejeon, Korea) between June 2018 and January 2019, 29 were enrolled for analysis (IRB No. 2018–06–021–002). Inclusion criteria were as follows: (a) age younger than 10 years with benign tonsillar hypertrophy, Friedman grading scale > 2, resulting in sleep apnea and/or mouth breathing and (b) patients who did not wear dentures or braces. Exclusion criteria were as follows: (a) recent (last 3 months) or frequent (3 times/year) history of tonsillitis, (b) systemic disease, (c) intake of antibiotics in the last 3 months, (d) a habit of regular oral rinse use and (e) breastfeeding.

Oral examination and sample collection were conducted at the Department of Otolaryngology-Head and Neck Surgery of CNUH. All participants remained fasted after midnight the day before until the completion of surgery according to the regular preoperative protocol.

Saliva was collected from the mouths of patients on the morning of surgery. Prior to sample collection, participants were instructed not to brush their teeth from the previous night to the time of sampling and were prohibited from eating or drinking for at least 9 h. In brief, participants were asked to chew on sterile gauze (4 × 4 inch) for 1 min to stimulate salivation before placing the gauze with absorbed saliva back into a sterile Eppendorf conical tube (50 mL). Tubes were centrifuged for 15 min at 1763 × *g* with a 100 μm cell strainer to collect clear saliva samples that were (immediately) stored at −70 °C until DNA extraction.

After general anesthesia, en bloc resection of both palatine tonsils via the peroral approach was performed according to the traditional tonsillectomy procedure using a cold knife and electrocautery. Approximately half the tissue around the upper pole of the palatine tonsils was sent to a pathological laboratory for clinical diagnosis, and the remainder was used for microbiome analysis.

### Anthropometric measurements and serum biochemical indices of subjects

We collected anthropometric data from all subjects, including the height and weight of all patients, which were measured the day before surgery. Body mass index (BMI) was calculated according to a previous report^15^. In addition, with reference to the 2017 Korean National Growth Chart, subjects were divided into two groups based on the 85^th^ percentile for BMI to determine obesity status.

Serum biochemical indices, including blood glucose, total cholesterol, and liver enzymes, such as aspartate aminotransferase (AST), alanine aminotransferase (ALT), white blood cell (WBC), red blood cell (RBC), and platelet (PLT) counts, were acquired from routine preoperative laboratory tests according to the guidelines of the American Society of Anesthesiologists (ASA).

### DNA extraction and amplicon sequencing

DNA from saliva and tonsillar tissue was extracted using a DNeasy PowerSoil kit (Qiagen, Germany). DNA in each sample was quantified using QIAxpert (Qiagen). Specific V3-V4 hypervariable regions of the 16S rRNA gene were amplified using 16S_V3_F (5′-TCGTCGGCAGCGTCAGATGTGTATAAGAGACAGCCTACGG GNGGCWGCAG-3′) and 16S_V4_R (5′-GTCTCGTGGGCTCGGAGATGTGTATA AGAGACAGGACTACHVGGGTATCTAATCC-3′) primers. Libraries were prepared using PCR products and quantified with the aid of the QIAxpert kit (Qiagen). Each amplicon was quantified, pooled, and sequenced using MiSeq (Illumina, USA). All raw sequences derived from this experiment were submitted to the Short Read Archive of NCBI and can be found under BioProject accession number #PRJNA615768.

### Analysis of bacterial compositions of microbiomes

A total of 726,274 raw reads were generated from the saliva and tonsillar tissue of 29 participants, with an average of 12,745 reads (standard deviation 3,859). Paired-end reads matching the adapter sequences were trimmed using cutadapt (version 1.1.6)^16^. The resulting FASTQ files containing paired-end reads were merged with CASPER and quality-filtered with a Phred (Q) score of 20^17^. Any reads <350 bp or >550 bp after merging were additionally excluded. To identify the chimeric sequences, a reference-based chimera detection step was conducted with VSEARCH against the SILVA gold database^18,19^. Next, sequence reads were clustered into OTUs using VSEARCH with a de novo clustering algorithm under 97% sequence similarity. The representative sequences of OTUs were finally classified using the SILVA 128 database with UCLUST (script on QIIME version 1.9.1) under default parameters^20^. Last, we then selected those that were detected in more than seven samples (over 25% of the total 29 patients) in the saliva and tonsil groups to ensure statistical reliability in the following analyses. We selected 1678 OTUs in the saliva group and 1461 OTUs in the tonsil group.

### Statistical analysis

Alpha diversities were calculated on a rarified dataset (3614 reads for saliva and 7833 reads for tonsillar tissue) using the QIIME pipeline. The alpha diversities for species richness and evenness (Chao1 index and Shannon index) were calculated. Statistical comparison of alpha diversities between tonsils and saliva was performed using the Kruskal–Wallis test, and *p* values were adjusted with Benjamini-Hochberg correction. The resultant distance matrix was applied to generate principal coordinate analysis (PCoA) plots based on the Bray-Curtis distance. The top 10 microbes ranked in terms of abundance were selected via Metastats analysis^21^. The Wilcoxon rank-sum test was additionally performed to compare the relative abundance of microbial taxa between saliva samples and tonsil samples.

Correlations of genera between saliva samples and tonsil samples were further analyzed using Spearman’s correlation coefficient. To identify microbial taxa, linear discriminant analysis (LDA) effect size (LEfSe), considering the statistical significance and biological relevance, was performed. The threshold of microbial taxa was identified as an LDA score > 2.5. All statistical analyses were performed using R version 3.6.1. *p* values < 0.05 were considered statistically significant.

## Results

### General characteristics and biochemical indices of subjects

A total of 29 healthy participants (23 boys and 6 girls) who had undergone tonsillectomy were enrolled. Table 1 presents the general and biochemical characteristics of the participants (left panel) along with the corresponding reference values for Korean children (right panel)^22,23^. The average anthropometric measurements were 7.66 ± 27.78 years for age, 128.52 ± 16.84 cm for height, 32.57 ± 16.28 kg for weight, and 18.59 ± 4.20 kg/m^2^ for BMI. Eleven participants were above the 85th percentile for BMI, and 18 were below the 85th percentile for BMI. Thus, 37.9% of participants were considered obese, and the remaining 62.1% belonged to the normal weight group. The average values of the biochemical indices from all participants were in the normal range for Korean children.

**Table 1:** General characteristics and biochemical indices of subjects.

Parameters	Total (Mean ± S.D.)	Korean children
Anthropometric measurement		
Boys	23	
Girls	6	
Age (years)	7.66 ± 2.78	6~12
Height (cm)	128.52 ± 16.84	114.7~151.7
Weight (kg)	32.57 ± 16.28	20.7~45.7
BMI (kg/m^2^)	18.59 ± 4.20	15.8~19.8
>85th percentile	11 (37.9%)	
<85th percentile	18 (62.1%)	
Biochemical indices		
Blood sugar (mg/dL)	91.72 ± 12.57	70~115
Total cholesterol (mg/dL)	166.67 ± 26.90	< 170
AST (U/L)	24.84 ± 10.84	15~40 (Boys), 13~35 (Girls)
ALT (U/L)	17.36 ± 10.68	10~35 (Boys), 10~30 (Girls)
WBC (×10^3^/mL)	7.21 ± 2.34	4.5~13.5
RBC (×10^6^/mL)	4.70 ± 0.26	4.2~6.0
PLT (×10^3^/mL)	299.16 ± 57.56	150~350

Anthropometric measurements and biochemical indices are presented as average values with standard deviations for subjects in this study (left panel), and the reference value is the range for Korean children (right panel).

### Total number of identified and classified microbes in saliva and tonsils

For comparative metagenomic analysis of the microbiomes between saliva samples and tonsil samples, DNA was extracted, and 16S rRNA gene sequencing was performed. Sequencing data were used to compare microbial communities between saliva samples and tonsil samples. A total of 726,274 raw reads were generated from the saliva and tonsillar tissue of 29 participants, with an average of 12,745 reads (standard deviation 3859). The taxonomic compositions of microbiota in saliva and tonsillar tissue were assigned based on the taxonomic level (Table 2). For taxon-based approaches, the sequences were identified as OTUs. In the saliva group, 9 phyla, 18 classes, 27 orders, 44 families, 97 genera, 125 species and 213 OTUs were annotated, while 9 phyla, 16 classes, 23 orders, 33 families, 69 genera, 95 species, and 212 OTUs were annotated in the tonsil group.

**Table 2:** Analysis of microbiomes of saliva and tonsillar tissues.

Total number of microbes identified	Phylum	Class	Order	Family	Genus	Species	OTUs
Saliva	9	18	27	44	97	125	213
Tonsils	9	16	23	33	69	95	212

Microbiome analysis was performed based on the V3–V4 hypervariable regions of 16S rRNA genes. Total number of microbes identified by taxonomic classification after OTU-based analysis.

### Microbiome composition at the genus level in saliva and tonsils

**Fig. 1:**
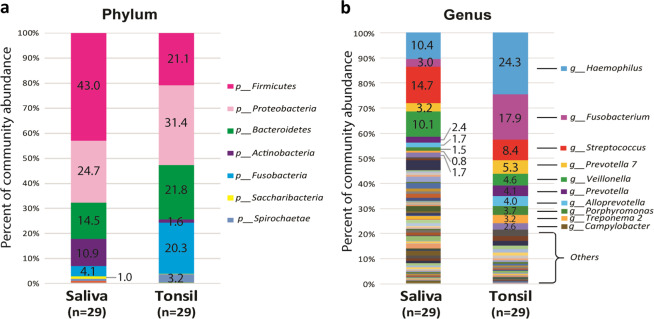
Microbiota compositions of saliva and tonsil samples from 29 participants. Relative abundance at **a** phylum level and **b** genus level from 29 participants. The relative abundance of the microbial community based on the dominant phyla and genera. The highly abundant phyla were *Firmicutes*, *Proteobacteria*, *Bacteroidetes, Actinobacteria*, and *Fusobacteria*. The predominant genera were *Haemophilus*, *Fusobacterium*, *Streptococcus*, *Prevotella* and *Veillonella*.

The microbiome compositions of saliva and tonsillar tissues were determined based on the average relative abundance assigned to the phylum and genus levels (Fig. 1). The saliva group showed a significantly higher abundance of *Firmicutes* (43.0%), followed by *Proteobacteria* (24.7%), *Bacteroidetes* (14.5%), *Actinobacteria* (10.9%) and *Fusobacteria* (4.1%) at the phylum level, while the tonsil group contained more *Proteobacteria* (31.4%), followed by *Bacteroidetes* (21.8%), *Firmicutes* (21.1%), *Fusobacteria* (20.3%) and *Spirochaetae* (3.2%). At the genus level, *Streptococcus* (14.7%) was the predominant taxon in the saliva group, and *Haemophilus* (24.3%) was the predominant taxon in the tonsil group.

### Comparison of alpha diversities

**Fig. 2:**
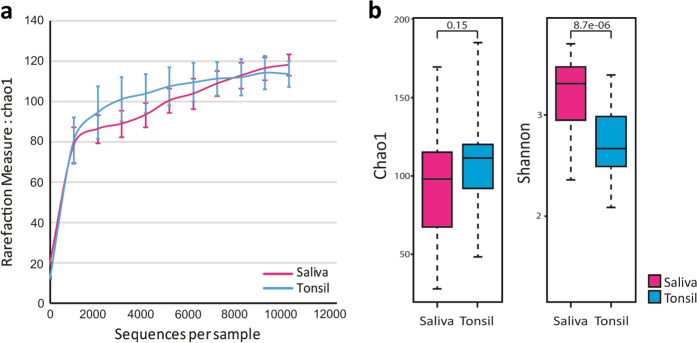
Comparison of alpha diversity between saliva and tonsils. **a** Rarefaction measured based on the Chao1 index and **b** box plots for alpha diversity based on the Chao1 and Shannon indices. Magenta represents saliva, and blue signifies tonsillar tissues.

We further assessed alpha diversity, which is indicative of species abundance, within each group. The alpha diversities of saliva and tonsils were measured based on the Chao1 index and species richness were compared. Rarefaction curves for the sequences per sample are shown in Fig. 2a. Chao1 and Shannon indices were employed to calculate the species diversity of samples between saliva samples and tonsil samples (Fig. 2b). The Chao1 index values, representing microbiome richness, in saliva and tonsils showed no significant differences (*p* = 0.15). On the other hand, the Shannon index values, representing the richness and the evenness of the distribution of the microbial community, in saliva were significantly higher than those in tonsillar tissue (*p* < 0.001). Our findings suggest that the bacterial community in the saliva is more evenly distributed than that in tonsils.

### Principal coordinate analysis (PCoA) biplots

**Fig. 3:**
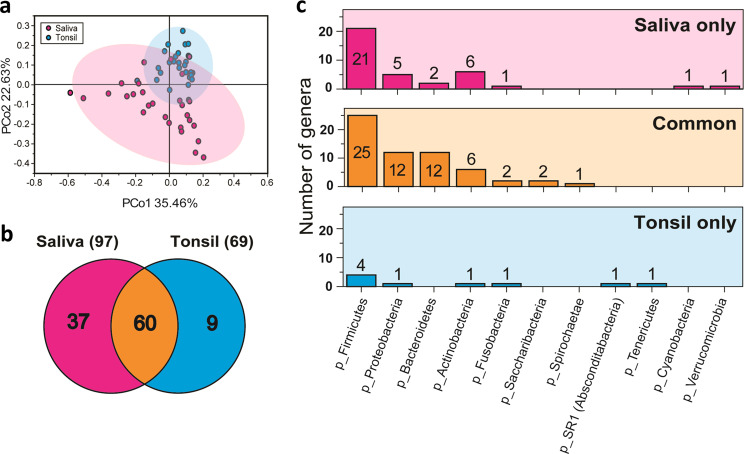
Comparison of saliva and tonsil groups at the genus level. Principal coordinate analysis plots of Bray-Curtis-computed distances between saliva and tonsils. **a** Saliva (red) and tonsil (blue) samples are colored according to the subject. The plot presents a 95% confidence ellipse with a color background. **b** Venn diagram showing the overall overlap between saliva and tonsils at the genus level. **c** Number of specific and common genera between saliva and tonsil samples at the phylum level.

Microbe communities from saliva and tonsillar tissues were discriminated based on principal coordinates analysis (PCoA). PCoA was used to compare community phylogenetic compositions and revealed microbial diversity between the groups. Based on PCo2 scores, tonsillar tissues were clearly distinguishable from saliva samples (Fig. 3a). A Venn diagram illustrated 97 genera in the saliva group and 69 in the tonsil group (Fig. 3b). Detailed information on common genera between the groups (saliva-specific and tonsil-specific) is listed in Supplementary Table 1. In total, 60 common genera were identified in both sample groups, corresponding to 61.9 and 87.0% of the 97 and 69 microbial genera determined in saliva and tonsils, respectively. Overall, 87.0% of the tonsil microbiome was identical to that of the oral saliva microbiome at the genus level, clearly signifying high similarities of the microbial communities of the two groups.

To determine bacterial distribution from the Venn diagram, a bar graph of all genera at the phylum level was generated (Fig. 3c). *Firmicutes*, *Actinobacteria*, *Proteobacteria*, *Bacteroidetes*, and *Fusobacteria* were abundant in saliva (upper panel), while in tonsillar tissue, the abundant phyla were *Firmicutes*, *Proteobacteria*, *Actinobacteria*, *Fusobacteria, SR1*, and *Tenericutes* (lower panel). Common phyla in both tissue types were *Firmicutes*, *Proteobacteria, Bacteroidetes, Actinobacteria, Fusobacteria, Saccharibacteria*, and *Spirochaetae* (middle panel).

### Metastats analysis of the differential abundance of the top 10 bacterial taxa in saliva and tonsil tissue samples

The top 10 ranked taxa in saliva and tonsillar tissue groups based on average relative abundance were *Haemophilus* (saliva (S): 10.4%, tonsils (T): 24.3%), *Streptococcus* (S: 14.7%, T: 8.4%), *Fusobacterium* (S: 3.0%, T: 17.9%), *Veillonella* (S: 10.1%, T: 4.6%), *Prevotella* (S: 2.4%, T: 4.1%), *Alloprevotella* (S: 1.7%, T: 4.0%), *Neisseria* (S: 3.9%, T: 1.8%), *Porphyromonas* (S: 1.5%, T: 3.7%), *Campylobacter* (S: 1.7%, T: 2.6%), and *Treponema 2* (S: 0.8%, T: 3.2%) (Table 3). The phyla of the top 10 ranked taxa were *Proteobacteria*, *Fusobacteria*, *Firmicutes*, *Bacteroidetes*, and *Spirochaetae*. Most identified genera included Gram-negative bacteria except *Streptococcus*. Moreover, 6 out of the top 10 ranked taxa were obligate anaerobes. However, the most abundant bacteria in tonsils and saliva were facultative anaerobes, such as *Haemophilus* and *Streptococcus*.

**Table 3:** Top 10 microbes ranked by Metastats analysis.

Order	Phylum	Genus	Saliva (%)	Tonsil (%)	Gram staining	Aerobic/anaerobic
1	Proteobacteria	*Haemophilus*	10.4	24.3	−	Facultative anaerobe
2	Firmicutes	*Streptococcus*	14.7	8.4	+	Facultative anaerobe
3	Fusobacteria	*Fusobacterium*	3.0	17.9	−	Obligate anaerobe
4	Firmicutes	*Veillonella*	10.1	4.6	−	Obligate anaerobe
5	Bacteriodetes	*Prevotella*	2.4	4.1	−	Obligate anaerobe
6	Bacteriodetes	*Alloprevotella*	1.7	4.0	−	Obligate anaerobe
7	Proteobacteria	*Neisseria*	3.9	1.8	−	Facultative anaerobe
8	Bacteriodetes	*Porphyromonas*	1.5	3.7	−	Obligate anaerobe
9	Proteobacteria	*Campylobacter*	1.7	2.6	−	Microaerophilic
10	Spirochaetae	*Treponema* 2	0.8	3.2	−	Obligate anaerobe

Metastats analysis of the abundance of bacterial taxa detected in saliva and tonsil tissue samples from the two groups.

### Correlation plots of individual microbes in saliva and tonsils

**Fig. 4:**
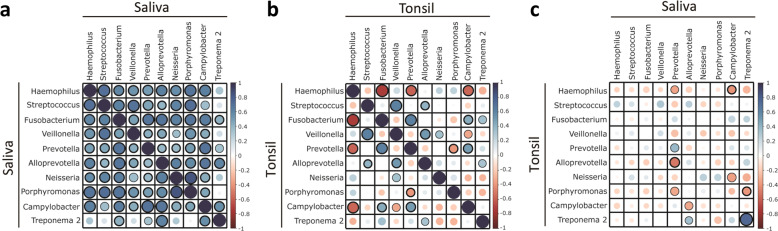
Correlation of the top 10 ranked microbes. Correlation plots of the top 10 ranked microbes **a** within the saliva, **b** within tonsils, and **c** between saliva and tonsils. A correlation matrix plot (based on Spearman’s correlation) is presented. Positive and negative correlations are represented by blue or red circles, respectively, and the size and color of circles refer to the correlation value. Significant correlations are indicated by the black outline of the circle with *p* ≤ 0.05 (thick border) and *p* ≤ 0.1 (thin border).

Spearman’s correlation analysis was applied to compare microbiomes within saliva and tonsillar tissues as well as between saliva and tonsils for the top 10 ranked taxa (Fig. 4). In general, the top 10 ranked microbiota within saliva showed high positive correlations with each other (Fig. 4a), while the top 10 ranked microbes within tonsils displayed both positive and negative correlations (Fig. 4b). *Haemophilus* and *Fusobacterium* within the tonsil group showed the strongest negative correlation with each other (*r* = −0.73, *p* < 0.001). For the correlation between tonsils and saliva, only two were statistically significant (*Treponema 2* and *Prevotella*), and all the bacterial taxa showed a positive correlation except one, suggesting high similarities between the two bacterial communities. Specifically, *Treponema 2* showed the strongest positive correlation between saliva and tonsils (*r* = 0.75, *p* < 0.001), while the strongest negative correlation was found between *Prevotella* in saliva and *Alloprevotella* in tonsillar tissues (*r* = −0.51, *p* < 0.01) and between *Campylobacter* in saliva and *Haemophilus* in tonsils (*r* = −0.41, *p* < 0.05) (Fig. 4c). Supplementary Table 2 presents the correlation coefficient scores and *p* values within saliva and within tonsillar tissues and between saliva and tonsils. Microbes with high correlations (*p* < 0.1) included *Prevotella*, *Alloprevotella*, *Campylobacter*, and *Treponema 2* in saliva and *Haemophilus*, *Prevotella*, *Alloprevotella*, *Neisseria*, *Porphyromonas*, *Campylobacter*, and *Treponema 2* in tonsils. *Prevotella* in tonsils showed significant negative correlations with *Haemophilus* (*r* = −0.35), *Alloprevotella* (*r* = −0.51), and *Porphyromonas* (*r* = −0.34) in saliva. A significant positive correlation was found between saliva and tonsils for *Prevotella* (*r* = 0.37).

### Taxonomic-level comparison of microbiota in saliva and tonsils

**Fig. 5:**
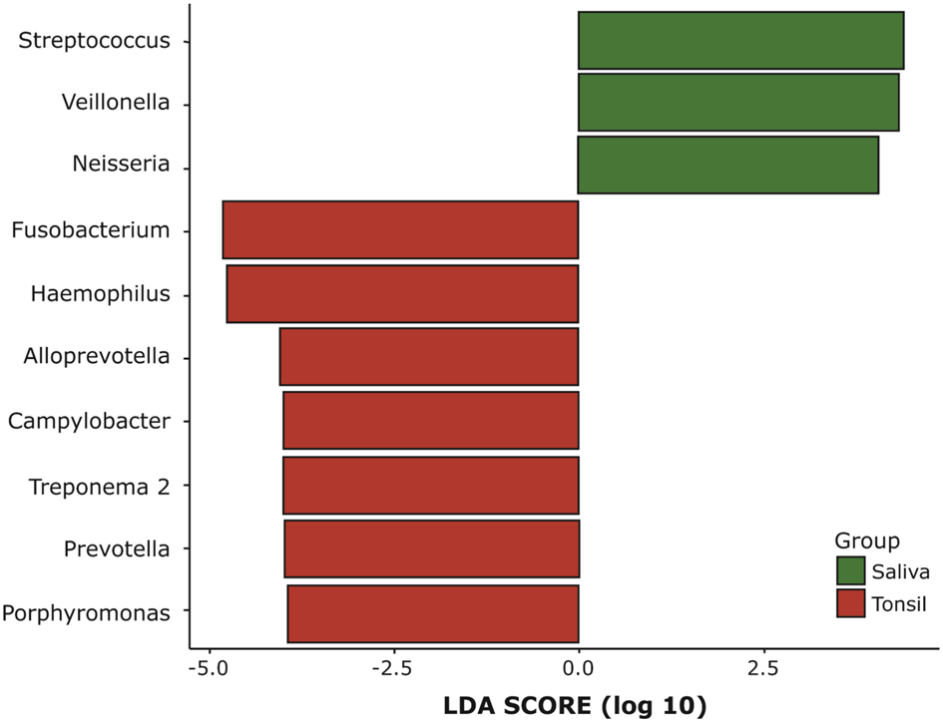
LDA effect size (LEfSe) analysis of tonsils compared to saliva for the top 10 ranked genera. LEfSe analysis was used to select microbiota that was significantly more abundant in one group, while the bar size represents the size of the effect. Green bars represent microbiota associated with saliva, while red bars represent tonsil-associated microbiota.

Linear discriminant analysis (LDA) effect size (LEfSe) analysis was used to identify significantly different saliva and tonsil microbiota compositions for the top 10 ranked taxa (Fig. 5). The threshold of the logarithmic LDA score for discriminative features was > 2.5. The enriched genera in the saliva group were *Streptococcus*, *Veillonella*, and *Neisseria*, and those in the tonsil group were *Fusobacterium*, *Haemophilus*, *Alloprevotella*, *Campylobacter*, *Treponema 2*, *Prevotella*, and *Porphyromonas*.

## Discussion

To our knowledge, this is the first study to compare the core microbiomes of saliva and tonsils in Korean pediatric patients without tonsillitis. By employing 16S rRNA gene sequencing, we determined the relative abundance of the microbial community in saliva and tonsils, with a view to establishing the association between the oral saliva microbial community and the tonsil microbiome profile in Korean children (Supplementary Fig. 1). Our data suggest that the microbiome profiles of saliva and tonsils are largely similar (87.0%), suggestive of interactions between their microbial components. To date, most studies on pediatric tonsils have reported on the tonsil microbiomes under inflammatory conditions such as tonsillitis. Our study is distinct from these previous studies in that we examined the microbiomes of saliva and tonsils in the absence of tonsillitis. Nevertheless, our study could not elucidate the microbiome of perfectly healthy tonsils because the tonsil microbiota samples used in this study were obtained from patients diagnosed with tonsillar hypertrophy. However, because children with normal tonsils do not need to undergo tonsillectomy, it is almost impossible to obtain perfectly normal tonsillar tissues.

Since tonsils have emerged as a good source of adult stem cells in tissue engineering and regenerative medicine, with the added advantages of noninvasive tissue collection^24^, relatively high proliferation rate and low allogenicity^25,26^, our research may facilitate the development of microbiome-based tools to regulate tonsil-derived therapeutic platforms. Further studies on larger groups are warranted to confirm initial findings.

Recent advances in sequencing technology and metagenomics have expanded our knowledge of the composition and association of the oral microbiome with human health and disease. The oral cavity microbiome has generally been examined through the collection of oral rinse samples, including saliva, but it remains to be established whether saliva provides an accurate representation of the microbiome of the oral cavity^27,28^. Given that the human oral cavity is composed of various subsites, including the teeth, gingival sulcus, tongue, hard and soft palates, and tonsils, which provide appropriate space for colonization but slightly different environments for microorganisms, the salivary microbiome profile may not be correlated with those of other subsites of the oral cavity.

Although a number of differences in the microbial profiles between recurrent tonsillitis and tonsillar hypertrophy pediatric groups have been determined using culture-based analysis, these experiments were limited in that they included only aerobic bacteria existing in the tonsillar core, and many human-associated microorganisms are not cultivable under laboratory settings^13^. Accordingly, we aimed to elucidate and compare the core microbiome profiles from paired saliva-tonsil samples using metagenome-wide analysis.

We obtained an average of 12,745 raw read counts, which was markedly lower than the 80,829 raw read count average reported in another study^29^. However, Caporaso and coworkers^30^ demonstrated that 2000 reads are sufficient to resolve relationships among samples observed with the full dataset. Accordingly, we considered our data adequate to determine microbiome correlations between saliva and tonsils. Our dataset, identified from 12,745 reads, included 213 OTUs (97 genera) in saliva and 212 OTUs (60 genera) in tonsils, which were reasonable counts for further analysis^31^.

Alpha diversity indices, including the Chao1 and Shannon–Weaver diversity indices, are generally used to compare the differences between microbial communities^32–34^. Both saliva and tonsils showed similar richness in microbial communities, but the saliva microbiome presented higher evenness of distribution than that of tonsillar tissue. The microbiome profile of saliva appears to be more stable than that of tonsils. Saliva is constantly secreted from salivary glands into the oral cavity, containing a diverse bacterial population, while the microbial community in tonsils is mainly colonized in the recessed epithelium of deeply branched crypts^27,35^. Therefore, saliva continuously secreted from various oral sites contains diverse bacteria, but the range in tonsils is lower since the microbial populations stagnate in the crypts and remain limited.

Pyrosequencing analysis of saliva microbes in healthy Chinese children and adults revealed *Streptococcus*, *Prevotella*, *Neisseria*, *Haemophilus*, *Porphyromonas*, *Gemella*, *Rothia*, *Granulicatella*, *Fusobacterium*, *Actinomyces*, *Veillonella*, and *Aggregatibacter* as major components of the healthy saliva microbiome^36^. In our study, *Streptococcus* was the most abundant component, followed by *Haemophilus*, *Veillonella*, *Neisseria*, *Fusobacterium*, and *Prevotella* in saliva.

In a study on bacterial distribution in tonsils of Korean children, Jeong et al.^13^ isolated 966 microbes from the tonsil cores of 824 recurrent tonsillitis and tonsillar hypertrophy patients using the cultivation method. In their experiments, *H. influenzae* (31.4%) was most commonly isolated from cases of tonsillar hypertrophy, followed by *S. pyogenes* (24.2%), *S. aureus* (22.9%) and *S. pneumoniae* (12.6%). Similar to these earlier findings, *H. influenzae* was the dominant pathogen in the tonsillar hypertrophy group in our study. The microbiomes of tonsillar crypts in children and adults affected by recurrent tonsillitis were compared with those of healthy adults and children with tonsillar hyperplasia^29^. Twelve genera of microbial communities were identified in all samples regardless of age and health status. Notably, *H. influenzae*, *Neisseria* species and *S. pseudopneumoniae* were significantly more abundant in children. In our experiments, *Haemophilus* (24.3%) was the most prevalent bacterial species in tonsillar tissue, followed by *Fusobacterium* (17.9%) and *Streptococcus* (8.4%). Thus, *Haemophilus* has been identified as the most common bacterium, not only in recurrent tonsillitis but also in hypertrophic tonsil samples^8,9,13,37^.

*Haemophilus* and *Staphylococcus* are the representative microbiota in both tonsillectomized and nontonsillectomized children with recurrent and tonsillar hypertrophy^8,37^. *Haemophilus* was identified as the most common bacterial species in both saliva and tonsils in our study (Table 3). However, *Staphylococcus*, which is frequently detected in tonsillitis, was less abundant in our study and was weakly correlated between the groups (data not shown). Specifically, *Staphylococcus* was ranked 23rd in terms of abundance in tonsils and 30th in saliva. The low abundance of *Staphylococcus* may be explained by the fact that the bacterium is mainly associated with inflammation, and we selected only hyperplasia cases for analysis (excluding tonsillitis).

The predominant aerobic and facultative organisms were *H. influenzae*, followed by *Neisseria* and *S. aureus*, in pediatric patients subjected to tonsillectomy, while the predominant anaerobic bacteria were *Fusobacterium*, *Bacteroides* and *Prevotella melaninogenica*^10^. These earlier results were identical to our finding that *Haemophilus*, *Fusobacterium*, *Prevotella*, and *Neisseria* are among the top 10 ranked genera in tonsil and saliva samples from Korean children (Table 3). Among the top 10 microbiota identified in the current study, the dominant bacteria in both saliva and tonsils were facultative anaerobes. Moreover, the most abundant species in saliva (*Streptococcus* of *Firmicutes* phylum) and tonsils (*Haemophilus* of *Proteobacteria* phylum) were consistent with those of the previous reports^10,36,38,39^.

The top 10 ranked microbes in our study mostly showed a strong positive correlation within the saliva microbiome, while both positive and negative correlations were detected within the tonsil microbiome. These differences could be explained as follows: since saliva is continuously secreted and renewed in the oral cavity, the microbial community is relatively constant. Compared with saliva, microbiota in tonsils are exposed to an environment with an insufficient supply of oxygen and nutrients due to stagnation in the crypt, potentially leading to competition between two microbes and, consequently, a negative correlation.

According to Spearman’s ranking correlation analysis, *Treponema 2*, the causative bacterium of periodontitis^40^, showed the most significant positive correlation between the environments of the saliva and tonsils (Fig. 4c), although its abundance was low (0.8% in saliva, 3.2% in tonsils; Table 3). The genus *Treponema* contains both pathogenic (treponematoses) and nonpathogenic species. Nonpathogenic treponemes form part of the normal microbial flora of the oral cavity, intestinal tract and genital tract. A number of oral treponemes have been associated with gingivitis and periodontal disease^41^. *Prevotella* in saliva was negatively correlated with *Haemophilus*, *Alloprevotella*, and *Porphyromonas* in tonsils. *Prevotella* is one of the major bacteria causing periodontal diseases, such as periodontitis and gingivitis. Intriguingly, we identified another interesting correlation in this study. *Campylobacter* in saliva was negatively correlated with *Haemophilus* in tonsils. Previously, a meta-analysis suggested that tonsillectomy is associated with an increased risk of developing Crohn’s disease^42^, which is a major form of inflammatory bowel disease with a high prevalence of *Campylobacter*, particularly in pediatric patients^43^. It remains unclear why tonsillectomy is associated with an increased risk for developing this specific inflammatory bowel disease, but it seems that the negative correlation between *Campylobacter* and *Haemophilus* in our data could be considered as one possible explanation. These relations support links among dental inflammation, oropharyngeal inflammatory diseases, and inflammatory bowel disease, further indicating a possible association between the oral cavity (saliva) and oropharynx (tonsil) and the gut.

Based on the collective results, we have expanded our understanding of the interactions between the microbiomes of saliva and tonsils. Several factors are responsible for the loss of microbial diversity and homeostatic function, including inflammation, diet, xenobiotics, and altered host cell function^44^. Given its critical roles in multiple diseases, the microbiome has become an extremely attractive target for therapeutic interventions^45^. One of the biggest hurdles in microbiome research is the identification of cause-effect relationships and the design of microbiome-based therapies that are able to achieve predictable effects on the microbial community and host cell function. Further studies are required to develop strategies aimed at modulating the microbiome profile to improve host function and health.

In conclusion, the analysis of the microbiomes of paired tonsil and saliva samples from Korean pediatric patients diagnosed with hyperplasia without tonsillitis revealed that many bacterial communities are shared and show similarities in terms of diversity and composition, suggesting close interactions between the two microbial groups. Although further studies are clearly essential, we assume that the oral microbiome exerts significant effects on not only the tonsil itself but also the tonsil-derived immune or stem cells by regulating the microbial community^46,47^. Our preliminary study may serve as a cornerstone in that it sheds light on the possibility of future research on novel microbiome-based therapeutic tactics using tonsil-derived cells or a tonsil-related microenvironment.

## Supplementary information

supplementary information
