# Supplementary material for: Association between the microbiomes of tonsil and saliva samples isolated from pediatric patients subjected to tonsillectomy for the treatment of tonsillar hyperplasia
Source: Exp Mol Med. 2020 Sep 4;52(9):1564–73. doi: 10.1038/s12276-020-00487-6 (PMC8080726; doi:10.1038/s12276-020-00487-6)
Supplement: Supplementary file 1 — supplementary information [file 12276_2020_487_MOESM1_ESM.pdf]

## Supplementary Fig. 1. Overview of the experimental framework

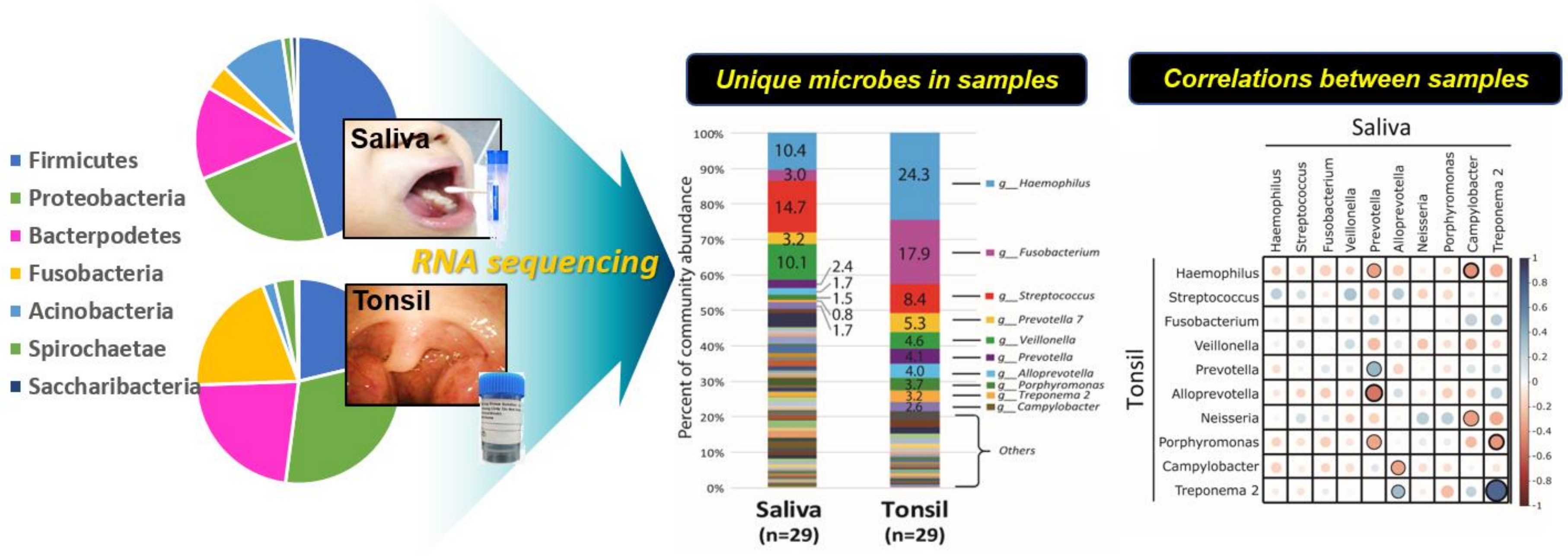

**Supplementary Table 1**. List of commonly annotated, saliva-specific, and tonsil-specific genera.

| | | |
|---|---|---|
| **Commonly annotated (60)** | 1 | k_Bacteria;p_Firmicutes;c_Bacilli;o_Lactobacillales;k_Bacteria_o_Lactobacillales;g_Streptococcus |
| | 2 | k_Bacteria;p_Proteobacteria;c_Gammaproteobacteria;o_Pasteurellales;k_Bacteria_o_Pasteurellales;g_Haemophilus |
| | 3 | k_Bacteria;p_Firmicutes;c_Negativicutes;o_Selenomonadales;k_Bacteria_o_Selenomonadales;g_Veillonella |
| | 4 | k_Bacteria;p_Proteobacteria;c_Betaproteobacteria;o_Neisseriales;k_Bacteria_o_Neisseriales;g_Neisseria |
| | 5 | k_Bacteria;p_Fusobacteria;c_Fusobacteriia;o_Fusobacteriales;k_Bacteria_o_Fusobacteriales;g_Fusobacterium |
| | 6 | k_Bacteria;p_Bacteroidetes;c_Bacteroidia;o_Bacteroidales;k_Bacteria_o_Bacteroidales;g_Prevotella 7 |
| | 7 | k_Bacteria;p_Bacteroidetes;c_Bacteroidia;o_Bacteroidales;k_Bacteria_o_Bacteroidales;g_Prevotella |
| | 8 | k_Bacteria;p_Actinobacteria;c_Actinobacteria;o_Micrococcales;k_Bacteria_o_Micrococcales;g_Rothia |
| | 9 | k_Bacteria;p_Actinobacteria;c_Actinobacteria;o_Actinomycetales;k_Bacteria_o_Actinomycetales;g_Actinomyces |
| | 10 | k_Bacteria;p_Firmicutes;c_Negativicutes;o_Selenomonadales;k_Bacteria_o_Selenomonadales;g_Selenomonas 3 |
| | 11 | k_Bacteria;p_Firmicutes;c_Clostridia;o_Clostridiales;k_Bacteria_o_Clostridiales;g_Faecalibacterium |
| | 12 | k_Bacteria;p_Bacteroidetes;c_Bacteroidia;o_Bacteroidales;k_Bacteria_o_Bacteroidales;g_Prevotella 9 |
| | 13 | k_Bacteria;p_Bacteroidetes;c_Bacteroidia;o_Bacteroidales;k_Bacteria_o_Bacteroidales;g_Alloprevotella |
| | 14 | k_Bacteria;p_Proteobacteria;c_Epsilonproteobacteria;o_Campylobacterales;k_Bacteria_o_Campylobacterales;g_Campylobacter |
| | 15 | k_Bacteria;p_Bacteroidetes;c_Bacteroidia;o_Bacteroidales;k_Bacteria_o_Bacteroidales;g_uncultured |
| | 16 | k_Bacteria;p_Firmicutes;c_Clostridia;o_Clostridiales;k_Bacteria_o_Clostridiales;g_Ruminococcaceae UCG-014 |
| | 17 | k_Bacteria;p_Bacteroidetes;c_Bacteroidia;o_Bacteroidales;k_Bacteria_o_Bacteroidales;g_Porphyromonas |
| | 18 | k_Bacteria;p_Actinobacteria;c_Actinobacteria;o_Bifidobacteriales;k_Bacteria_o_Bifidobacteriales;g_Bifidobacterium |
| | 19 | k_Bacteria;p_Firmicutes;c_Bacilli;o_Lactobacillales;k_Bacteria_o_Lactobacillales;g_Granulicatella |
| | 20 | k_Bacteria;p_Proteobacteria;c_Gammaproteobacteria;o_Pseudomonadales;k_Bacteria_o_Pseudomonadales;g_Pseudomonas |
| | 21 | k_Bacteria;p_Saccharibacteria;c_uncultured bacterium;o_uncultured bacterium;k_Bacteria_o_uncultured bacterium;g_uncultured bacterium |
| | 22 | k_Bacteria;p_Actinobacteria;c_Actinobacteria;o_Corynebacteriales;k_Bacteria_o_Corynebacteriales;g_Corynebacterium 1 |
| | 23 | k_Bacteria;p_Proteobacteria;c_Betaproteobacteria;o_Burkholderiales;k_Bacteria_o_Burkholderiales;g_Burkholderia-Paraburkholderia |
| | 24 | k_Bacteria;p_Firmicutes;c_Bacilli;o_Bacillales;k_Bacteria_o_Bacillales;g_Staphylococcus |
| | 25 | k_Bacteria;p_Firmicutes;c_Bacilli;o_Bacillales;k_Bacteria_o_Bacillales;g_Gemella |
| | 26 | k_Bacteria;p_Spirochaetae;c_Spirochaetes;o_Spirochaetales;k_Bacteria_o_Spirochaetales;g_Treponema 2 |
| | 27 | k_Bacteria;p_Proteobacteria;c_Gammaproteobacteria;o_Pasteurellales;k_Bacteria_o_Pasteurellales;g_Actinobacillus |
| | 28 | k_Bacteria;p_Firmicutes;c_Negativicutes;o_Selenomonadales;k_Bacteria_o_Selenomonadales;g_Dialister |
| | 29 | k_Bacteria;p_Proteobacteria;c_Betaproteobacteria;o_Burkholderiales;k_Bacteria_o_Burkholderiales;g_Lautropia |
| | 30 | k_Bacteria;p_Fusobacteria;c_Fusobacteriia;o_Fusobacteriales;k_Bacteria_o_Fusobacteriales;g_Leptotrichia |
| | 31 | k_Bacteria;p_Firmicutes;c_Clostridia;o_Clostridiales;k_Bacteria_o_Clostridiales;g_Peptoclostridium |
| | 32 | k_Bacteria;p_Firmicutes;c_Clostridia;o_Clostridiales;k_Bacteria_o_Clostridiales;g_Parvimonas |
| | 33 | k_Bacteria;p_Firmicutes;c_Negativicutes;o_Selenomonadales;k_Bacteria_o_Selenomonadales;g_Selenomonas |
| | 34 | k_Bacteria;p_Bacteroidetes;c_Flavobacteriia;o_Flavobacteriales;k_Bacteria_o_Flavobacteriales;g_Capnocytophaga |
| | 35 | k_Bacteria;p_Proteobacteria;c_Betaproteobacteria;o_Neisseriales;k_Bacteria_o_Neisseriales;g_Kingella |
| | 36 | k_Bacteria;p_Proteobacteria;c_Gammaproteobacteria;o_Pasteurellales;k_Bacteria_o_Pasteurellales;g_Aggregatibacter |
| | 37 | k_Bacteria;p_Firmicutes;c_Clostridia;o_Clostridiales;k_Bacteria_o_Clostridiales;g_Oribacterium |
| | 38 | k_Bacteria;p_Proteobacteria;c_Betaproteobacteria;o_Burkholderiales;k_Bacteria_o_Burkholderiales;g_Ralstonia |
| | 39 | k_Bacteria;p_Firmicutes;c_Clostridia;o_Clostridiales;k_Bacteria_o_Clostridiales;g_[Eubacterium] brachy group |
| | 40 | k_Bacteria;p_Firmicutes;c_Clostridia;o_Clostridiales;k_Bacteria_o_Clostridiales;g_Blautia |
| | 41 | k_Bacteria;p_Firmicutes;c_Clostridia;o_Clostridiales;k_Bacteria_o_Clostridiales;g_Lachnospiraceae UCG-008 |
| | 42 | k_Bacteria;p_Bacteroidetes;c_Bacteroidia;o_Bacteroidales;k_Bacteria_o_Bacteroidales;g_Prevotella 6 |
| | 43 | k_Bacteria;p_Firmicutes;c_Clostridia;o_Clostridiales;k_Bacteria_o_Clostridiales;g_Lachnoanaerobaculum |
| | 44 | k_Bacteria;p_Actinobacteria;c_Actinobacteria;o_Corynebacteriales;k_Bacteria_o_Corynebacteriales;g_Corynebacterium |
| | 45 | k_Bacteria;p_Bacteroidetes;c_Bacteroidia;o_Bacteroidales;k_Bacteria_o_Bacteroidales;g_Bacteroides |
| | 46 | k_Bacteria;p_Actinobacteria;c_Coriobacteriia;o_Coriobacteriales;k_Bacteria_o_Coriobacteriales;g_Atopobium |
| | 47 | k_Bacteria;p_Firmicutes;c_Bacilli;o_Lactobacillales;k_Bacteria_o_Lactobacillales;g_Abiotrophia |
| | 48 | k_Bacteria;p_Bacteroidetes;c_Bacteroidia;o_Bacteroidales;k_Bacteria_o_Bacteroidales;g_Prevotella 2 |
| | 49 | k_Bacteria;p_Bacteroidetes;c_Flavobacteriia;o_Flavobacteriales;k_Bacteria_o_Flavobacteriales;g_Bergeyella |
| | 50 | k_Bacteria;p_Firmicutes;c_Clostridia;o_Clostridiales;k_Bacteria_o_Clostridiales;g_Johnsonella |
| | 51 | k_Bacteria;p_Firmicutes;c_Erysipelotrichia;o_Erysipelotrichales;k_Bacteria_o_Erysipelotrichales;g_Erysipelotrichaceae UCG-007 |
| | 52 | k_Bacteria;p_Firmicutes;c_Clostridia;o_Clostridiales;k_Bacteria_o_Clostridiales;g_[Eubacterium] nodatum group |
| | 53 | k_Bacteria;p_Firmicutes;c_Clostridia;o_Clostridiales;k_Bacteria_o_Clostridiales;g_Catonella |
| | 54 | k_Bacteria;p_Bacteroidetes;c_Bacteroidia;o_Bacteroidales;k_Bacteria_o_Bacteroidales;g_Tannerella |
| | 55 | k_Bacteria;p_Firmicutes;c_Clostridia;o_Clostridiales;k_Bacteria_o_Clostridiales;g_Stomatobaculum |
| | 56 | k_Bacteria;p_Firmicutes;c_Clostridia;o_Clostridiales;k_Bacteria_o_Clostridiales;g_Peptococcus |
| | 57 | k_Bacteria;p_Saccharibacteria;c_Unknown Class;o_Unknown Order;k_Bacteria_o_Unknown Order;g_Candidatus Saccharimonas |
| | 58 | k_Bacteria;p_Proteobacteria;c_Gammaproteobacteria;o_Cardiobacteriales;k_Bacteria_o_Cardiobacteriales;g_Cardiobacterium |
| | 59 | k_Bacteria;p_Proteobacteria;c_Betaproteobacteria;o_Neisseriales;k_Bacteria_o_Neisseriales;g_Eikenella |
| | 60 | k_Bacteria;p_Firmicutes;c_Clostridia;o_Clostridiales;k_Bacteria_o_Clostridiales;g_Mogibacterium |
| | 1 | k_Bacteria;p_Actinobacteria;c_Actinobacteria;o_Micrococcales;k_Bacteria_o_Micrococcales;g_Kocuria |
| | 2 | k_Bacteria;p_Proteobacteria;c_Betaproteobacteria;o_Neisseriales;k_Bacteria_o_Neisseriales;g_uncultured |
| | 3 | k_Bacteria;p_Proteobacteria;c_Gammaproteobacteria;o_Enterobacteriales;k_Bacteria_o_Enterobacteriales;g_Escherichia-Shigella |
| | 4 | k_Bacteria;p_Actinobacteria;c_Coriobacteriia;o_Coriobacteriales;k_Bacteria_o_Coriobacteriales;g_Collinsella |
| | 5 | k_Bacteria;p_Firmicutes;c_Clostridia;o_Clostridiales;k_Bacteria_o_Clostridiales;g_Subdoligranulum |
| | 6 | k_Bacteria;p_Proteobacteria;c_Gammaproteobacteria;o_Pseudomonadales;k_Bacteria_o_Pseudomonadales;g_Acinetobacter |

| | | |
|---|---|---|
| **Saliva-specific annotated (37)** | 7 | k_Bacteria;p_Verrucomicrobia;c_Verrucomicrobiae;o_Verrucomicrobiales;k_Bacteria_o_Verrucomicrobiales;g_Akkermansia |
| | 8 | k_Bacteria;p_Firmicutes;c_Clostridia;o_Clostridiales;k_Bacteria_o_Clostridiales;g_[Eubacterium] rectale group |
| | 9 | k_Bacteria;p_Actinobacteria;c_Actinobacteria;o_Propionibacteriales;k_Bacteria_o_Propionibacteriales;g_Propionibacterium |
| | 10 | k_Bacteria;p_Cyanobacteria;c_Chloroplast;o_Tectona grandis;k_Bacteria_o_Tectona grandis;g_Tectona grandis |
| | 11 | k_Bacteria;p_Firmicutes;c_Clostridia;o_Clostridiales;k_Bacteria_o_Clostridiales;g_Ruminococcaceae UCG-013 |
| | 12 | k_Bacteria;p_Bacteroidetes;c_Bacteroidia;o_Bacteroidales;k_Bacteria_o_Bacteroidales;g_Alistipes |
| | 13 | k_Bacteria;p_Bacteroidetes;c_Bacteroidia;o_Bacteroidales;k_Bacteria_o_Bacteroidales;g_uncultured bacterium |
| | 14 | k_Bacteria;p_Firmicutes;c_Clostridia;o_Clostridiales;k_Bacteria_o_Clostridiales;g_Clostridium sensu stricto 1 |
| | 15 | k_Bacteria;p_Firmicutes;c_Bacilli;o_Lactobacillales;k_Bacteria_o_Lactobacillales;g_Lactobacillus |
| | 16 | k_Bacteria;p_Firmicutes;c_Clostridia;o_Clostridiales;k_Bacteria_o_Clostridiales;g_[Ruminococcus] torques group |
| | 17 | k_Bacteria;p_Firmicutes;c_Erysipelotrichia;o_Erysipelotrichales;k_Bacteria_o_Erysipelotrichales;g_Holdemanella |
| | 18 | k_Bacteria;p_Firmicutes;c_Clostridia;o_Clostridiales;k_Bacteria_o_Clostridiales;g_Lachnospiraceae NK4A136 group |
| | 19 | k_Bacteria;p_Actinobacteria;c_Actinobacteria;o_Bifidobacteriales;k_Bacteria_o_Bifidobacteriales;g_Gardnerella |
| | 20 | k_Bacteria;p_Firmicutes;c_Clostridia;o_Clostridiales;k_Bacteria_o_Clostridiales;g_Peptostreptococcus |
| | 21 | k_Bacteria;p_Proteobacteria;c_Gammaproteobacteria;o_Enterobacteriales;k_Bacteria_o_Enterobacteriales;g_Salmonella |
| | 22 | k_Bacteria;p_Firmicutes;c_Clostridia;o_Clostridiales;k_Bacteria_o_Clostridiales;g_Ruminiclostridium 9 |
| | 23 | k_Bacteria;p_Proteobacteria;c_Alphaproteobacteria;o_Rickettsiales;k_Bacteria_o_Rickettsiales;g_Solanum melongena (eggplant) |
| | 24 | k_Bacteria;p_Fusobacteria;c_Fusobacteriia;o_Fusobacteriales;k_Bacteria_o_Fusobacteriales;g_uncultured bacterium |
| | 25 | k_Bacteria;p_Firmicutes;c_Clostridia;o_Clostridiales;k_Bacteria_o_Clostridiales;g_Ruminococcus 2 |
| | 26 | k_Bacteria;p_Firmicutes;c_Clostridia;o_Clostridiales;k_Bacteria_o_Clostridiales;g_Fusicatenibacter |
| | 27 | k_Bacteria;p_Firmicutes;c_Clostridia;o_Clostridiales;k_Bacteria_o_Clostridiales;g_Anaerostipes |
| | 28 | k_Bacteria;p_Firmicutes;c_Clostridia;o_Clostridiales;k_Bacteria_o_Clostridiales;g_Roseburia |
| | 29 | k_Bacteria;p_Actinobacteria;c_Actinobacteria;o_Micrococcales;k_Bacteria_o_Micrococcales;g_Micrococcus |
| | 30 | k_Bacteria;p_Firmicutes;c_Clostridia;o_Clostridiales;k_Bacteria_o_Clostridiales;g_[Eubacterium] hallii group |
| | 31 | k_Bacteria;p_Actinobacteria;c_Actinobacteria;o_Micrococcales;k_Bacteria_o_Micrococcales;g_Knoellia |
| | 32 | k_Bacteria;p_Firmicutes;c_Clostridia;o_Clostridiales;k_Bacteria_o_Clostridiales;g_[Eubacterium] coprostanoligenes group |
| | 33 | k_Bacteria;p_Firmicutes;c_Clostridia;o_Clostridiales;k_Bacteria_o_Clostridiales;g_Coprococcus 2 |
| | 34 | k_Bacteria;p_Firmicutes;c_Clostridia;o_Clostridiales;k_Bacteria_o_Clostridiales;g_Ruminiclostridium 6 |
| | 35 | k_Bacteria;p_Firmicutes;c_Clostridia;o_Clostridiales;k_Bacteria_o_Clostridiales;g_Lachnoclostridium |
| | 36 | k_Bacteria;p_Firmicutes;c_Bacilli;o_Lactobacillales;k_Bacteria_o_Lactobacillales;g_Enterococcus |
| | 37 | k_Bacteria;p_Firmicutes;c_Erysipelotrichia;o_Erysipelotrichales;k_Bacteria_o_Erysipelotrichales;g_Erysipelotrichaceae UCG-003 |
| **Tonsil-specific annotated (9)** | 1 | k_Bacteria;p_Fusobacteria;c_Fusobacteriia;o_Fusobacteriales;k_Bacteria_o_Fusobacteriales;g_uncultured |
| | 2 | k_Bacteria;p_Tenericutes;c_Mollicutes;o_Mycoplasmatales;k_Bacteria_o_Mycoplasmatales;g_Mycoplasma |
| | 3 | k_Bacteria;p_Firmicutes;c_Negativicutes;o_Selenomonadales;k_Bacteria_o_Selenomonadales;g_Megasphaera |
| | 4 | k_Bacteria;p_Firmicutes;c_Negativicutes;o_Selenomonadales;k_Bacteria_o_Selenomonadales;g_Anaeroglobus |
| | 5 | k_Bacteria;p_Firmicutes;c_Clostridia;o_Clostridiales;k_Bacteria_o_Clostridiales;g_Shuttleworthia |
| | 6 | k_Bacteria;p_Actinobacteria;c_Coriobacteriia;o_Coriobacteriales;k_Bacteria_o_Coriobacteriales;g_Slackia |
| | 7 | k_Bacteria;p_SR1 (Absconditabacteria);c_uncultured bacterium;o_uncultured bacterium;k_Bacteria_o_uncultured bacterium;g_uncultured bacterium |
| | 8 | k_Bacteria;p_Firmicutes;c_Clostridia;o_Clostridiales;k_Bacteria_o_Clostridiales;g_uncultured |
| | 9 | k_Bacteria;p_Proteobacteria;c_Betaproteobacteria;o_Neisseriales;k_Bacteria_o_Neisseriales;g_Alysiella |

**Supplementary Table 2.** Correlations and *p* values within saliva, within tonsils, and between saliva and tonsils for the top 10 genera.

| | | | | | | | | | | | | | |
|---|---|---|---|---|---|---|---|---|---|---|---|---|---|
| **a** Within saliva | **correlation** | | | **Saliva** | | | | | | | | | |
| | | | | **Haemophilus** | **Streptococcus** | **Fusobacterium** | **Veillonella** | **Prevotella** | **Alloprevotella** | **Neisseria** | **Porphyromonas** | **Campylobacter** | **Treponema 2** |
| | | **Saliva** | **Haemophilus** | 1.000 | 0.704 | 0.592 | 0.539 | 0.502 | 0.622 | 0.567 | 0.688 | 0.623 | 0.315 |
| | | | **Streptococcus** | 0.704 | 1.000 | 0.613 | 0.692 | 0.465 | 0.393 | 0.605 | 0.720 | 0.354 | 0.151 |
| | | | **Fusobacterium** | 0.592 | 0.613 | 1.000 | 0.482 | 0.680 | 0.689 | 0.621 | 0.688 | 0.639 | 0.411 |
| | | | **Veillonella** | 0.539 | 0.692 | 0.482 | 1.000 | 0.426 | 0.461 | 0.360 | 0.536 | 0.431 | 0.187 |
| | | | **Prevotella** | 0.502 | 0.465 | 0.680 | 0.426 | 1.000 | 0.378 | 0.379 | 0.493 | 0.699 | 0.339 |
| | | | **Alloprevotella** | 0.622 | 0.393 | 0.689 | 0.461 | 0.378 | 1.000 | 0.623 | 0.575 | 0.677 | 0.609 |
| | | | **Neisseria** | 0.567 | 0.605 | 0.621 | 0.360 | 0.379 | 0.623 | 1.000 | 0.840 | 0.437 | 0.299 |
| | | | **Porphyromonas** | 0.688 | 0.720 | 0.688 | 0.536 | 0.493 | 0.575 | 0.840 | 1.000 | 0.463 | 0.056 |
| | | | **Campylobacter** | 0.623 | 0.354 | 0.639 | 0.431 | 0.699 | 0.677 | 0.437 | 0.463 | 1.000 | 0.557 |
| | | | **Treponema 2** | 0.315 | 0.151 | 0.411 | 0.187 | 0.339 | 0.609 | 0.299 | 0.056 | 0.557 | 1.000 |
| | ***p*  value** | | | **Saliva** | | | | | | | | | |
| | | | | **Haemophilus** | **Streptococcus** | **Fusobacterium** | **Veillonella** | **Prevotella** | **Alloprevotella** | **Neisseria** | **Porphyromonas** | **Campylobacter** | **Treponema 2** |
| | | **Saliva** | **Haemophilus** | 0.000 | 0.000 | 0.001 | 0.003 | 0.006 | 0.000 | 0.002 | 0.000 | 0.000 | 0.103 |
| | | | **Streptococcus** | 0.000 | 0.000 | 0.001 | 0.000 | 0.013 | 0.039 | 0.001 | 0.000 | 0.065 | 0.443 |
| | | | **Fusobacterium** | 0.001 | 0.001 | 0.000 | 0.009 | 0.000 | 0.000 | 0.000 | 0.000 | 0.000 | 0.030 |
| | | | **Veillonella** | 0.003 | 0.000 | 0.009 | 0.000 | 0.024 | 0.013 | 0.060 | 0.003 | 0.022 | 0.341 |
| | | | **Prevotella** | 0.006 | 0.013 | 0.000 | 0.024 | 0.000 | 0.047 | 0.047 | 0.008 | 0.000 | 0.078 |
| | | | **Alloprevotella** | 0.000 | 0.039 | 0.000 | 0.013 | 0.047 | 0.000 | 0.000 | 0.001 | 0.000 | 0.001 |
| | | | **Neisseria** | 0.002 | 0.001 | 0.000 | 0.060 | 0.047 | 0.000 | 0.000 | 0.000 | 0.020 | 0.122 |
| | | | **Porphyromonas** | 0.000 | 0.000 | 0.000 | 0.003 | 0.008 | 0.001 | 0.000 | 0.000 | 0.013 | 0.777 |
| | | | **Campylobacter** | 0.000 | 0.065 | 0.000 | 0.022 | 0.000 | 0.000 | 0.020 | 0.013 | 0.000 | 0.002 |
| | | | **Treponema 2** | 0.103 | 0.443 | 0.030 | 0.341 | 0.078 | 0.001 | 0.122 | 0.777 | 0.002 | 0.000 |
| | **correlation** | | | **Tonsil** | | | | | | | | | |
| | | | | **Haemophilus** | **Streptococcus** | **Fusobacterium** | **Veillonella** | **Prevotella** | **Alloprevotella** | **Neisseria** | **Porphyromonas** | **Campylobacter** | **Treponema 2** |
| | | **Tonsil** | **Haemophilus** | 1.000 | -0.226 | -0.733 | 0.206 | -0.584 | 0.057 | 0.258 | 0.245 | -0.574 | -0.298 |
| | | | **Streptococcus** | -0.226 | 1.000 | 0.098 | 0.622 | 0.042 | 0.402 | 0.031 | 0.028 | -0.168 | 0.094 |
| | | | **Fusobacterium** | -0.733 | 0.098 | 1.000 | -0.213 | 0.626 | -0.014 | -0.081 | -0.229 | 0.431 | 0.340 |
| | | | **Veillonella** | 0.206 | 0.622 | -0.213 | 1.000 | -0.189 | 0.519 | 0.336 | 0.065 | -0.337 | -0.142 |
| | | | **Prevotella** | -0.584 | 0.042 | 0.626 | -0.189 | 1.000 | -0.184 | -0.092 | -0.396 | 0.498 | 0.178 |
| | | | **Alloprevotella** | 0.057 | 0.402 | -0.014 | 0.519 | -0.184 | 1.000 | 0.183 | 0.104 | -0.134 | 0.324 |
| | | | **Neisseria** | 0.258 | 0.031 | -0.081 | 0.336 | -0.092 | 0.183 | 1.000 | 0.154 | -0.282 | -0.281 |

| | | | | | | | | | | | | | |
|---|---|---|---|---|---|---|---|---|---|---|---|---|---|
| **b** Within tonsil | | | **Porphyromonas** | 0.245 | 0.028 | -0.229 | 0.065 | -0.396 | 0.104 | 0.154 | 1.000 | -0.197 | -0.288 |
| | | | **Campylobacter** | -0.574 | -0.168 | 0.431 | -0.337 | 0.498 | -0.134 | -0.282 | -0.197 | 1.000 | 0.063 |
| | | | **Treponema 2** | -0.298 | 0.094 | 0.340 | -0.142 | 0.178 | 0.324 | -0.281 | -0.288 | 0.063 | 1.000 |
| | ***p* value** | | | **Tonsil** | | | | | | | | | |
| | | | | **Haemophilus** | **Streptococcus** | **Fusobacterium** | **Veillonella** | **Prevotella** | **Alloprevotella** | **Neisseria** | **Porphyromonas** | **Campylobacter** | **Treponema 2** |
| | | **Tonsil** | **Haemophilus** | 0.000 | 0.247 | 0.000 | 0.293 | 0.001 | 0.771 | 0.186 | 0.209 | 0.002 | 0.124 |
| | | | **Streptococcus** | 0.247 | 0.000 | 0.620 | 0.000 | 0.831 | 0.034 | 0.875 | 0.889 | 0.394 | 0.633 |
| | | | **Fusobacterium** | 0.000 | 0.620 | 0.000 | 0.277 | 0.000 | 0.943 | 0.681 | 0.241 | 0.023 | 0.076 |
| | | | **Veillonella** | 0.293 | 0.000 | 0.277 | 0.000 | 0.337 | 0.005 | 0.080 | 0.741 | 0.080 | 0.472 |
| | | | **Prevotella** | 0.001 | 0.831 | 0.000 | 0.337 | 0.000 | 0.346 | 0.641 | 0.037 | 0.008 | 0.365 |
| | | | **Alloprevotella** | 0.771 | 0.034 | 0.943 | 0.005 | 0.346 | 0.000 | 0.352 | 0.598 | 0.495 | 0.093 |
| | | | **Neisseria** | 0.186 | 0.875 | 0.681 | 0.080 | 0.641 | 0.352 | 0.000 | 0.434 | 0.146 | 0.147 |
| | | | **Porphyromonas** | 0.209 | 0.889 | 0.241 | 0.741 | 0.037 | 0.598 | 0.434 | 0.000 | 0.316 | 0.137 |
| | | | **Campylobacter** | 0.002 | 0.394 | 0.023 | 0.080 | 0.008 | 0.495 | 0.146 | 0.316 | 0.000 | 0.752 |
| | | | **Treponema 2** | 0.124 | 0.633 | 0.076 | 0.472 | 0.365 | 0.093 | 0.147 | 0.137 | 0.752 | 0.000 |
| **c** Between saliva and tonsil | **correlation** | | | **Saliva** | | | | | | | | | |
| | | | | **Haemophilus** | **Streptococcus** | **Fusobacterium** | **Veillonella** | **Prevotella** | **Alloprevotella** | **Neisseria** | **Porphyromonas** | **Campylobacter** | **Treponema 2** |
| | | **Tonsil** | **Haemophilus** | -0.188 | -0.123 | -0.171 | -0.151 | -0.352 | -0.181 | -0.048 | -0.096 | -0.407 | -0.289 |
| | | | **Streptococcus** | 0.244 | 0.167 | -0.069 | 0.302 | -0.201 | 0.261 | -0.166 | -0.128 | 0.065 | 0.056 |
| | | | **Fusobacterium** | -0.039 | -0.067 | 0.077 | -0.056 | 0.188 | 0.052 | -0.025 | -0.034 | 0.227 | 0.251 |
| | | | **Veillonella** | 0.059 | 0.113 | 0.013 | 0.186 | -0.240 | 0.096 | -0.220 | -0.117 | -0.222 | -0.133 |
| | | | **Prevotella** | -0.114 | 0.056 | 0.114 | 0.081 | 0.372 | -0.155 | -0.025 | -0.091 | 0.130 | 0.132 |
| | | | **Alloprevotella** | -0.099 | -0.163 | -0.197 | -0.111 | -0.505 | 0.165 | -0.078 | -0.188 | -0.177 | 0.233 |
| | | | **Neisseria** | -0.032 | 0.166 | 0.125 | -0.159 | -0.165 | 0.033 | 0.252 | 0.241 | -0.361 | -0.311 |
| | | | **Porphyromonas** | -0.169 | -0.108 | -0.186 | -0.084 | -0.335 | 0.035 | 0.088 | 0.087 | -0.258 | -0.400 |
| | | | **Campylobacter** | -0.166 | -0.066 | -0.151 | -0.102 | 0.115 | -0.330 | -0.108 | -0.114 | 0.041 | -0.084 |
| | | | **Treponema 2** | -0.062 | -0.085 | 0.085 | 0.018 | -0.033 | 0.342 | -0.070 | -0.250 | 0.222 | 0.754 |
| | ***p* value** | | | **Saliva** | | | | | | | | | |
| | | | | **Haemophilus** | **Streptococcus** | **Fusobacterium** | **Veillonella** | **Prevotella** | **Alloprevotella** | **Neisseria** | **Porphyromonas** | **Campylobacter** | **Treponema 2** |
| | | | **Haemophilus** | 0.337 | 0.531 | 0.386 | 0.444 | 0.066 | 0.356 | 0.807 | 0.628 | 0.031 | 0.136 |
| | | | **Streptococcus** | 0.212 | 0.395 | 0.726 | 0.118 | 0.306 | 0.179 | 0.399 | 0.518 | 0.742 | 0.778 |
| | | | **Fusobacterium** | 0.842 | 0.736 | 0.698 | 0.778 | 0.339 | 0.794 | 0.901 | 0.865 | 0.245 | 0.197 |
| | | | **Veillonella** | 0.766 | 0.569 | 0.949 | 0.344 | 0.218 | 0.626 | 0.261 | 0.554 | 0.257 | 0.499 |
| | | | **Prevotella** | 0.563 | 0.777 | 0.563 | 0.683 | 0.051 | 0.432 | 0.901 | 0.646 | 0.508 | 0.504 |

| | | | | | | | | | | | | | |
|---|---|---|---|---|---|---|---|---|---|---|---|---|---|
| | | Tonsil | **Alloprevotella** | 0.615 | 0.406 | 0.315 | 0.573 | 0.006 | 0.401 | 0.693 | 0.338 | 0.368 | 0.232 |
| | | | **Neisseria** | 0.871 | 0.397 | 0.526 | 0.418 | 0.402 | 0.869 | 0.196 | 0.216 | 0.059 | 0.107 |
| | | | **Porphyromonas** | 0.390 | 0.584 | 0.344 | 0.671 | 0.081 | 0.860 | 0.658 | 0.661 | 0.184 | 0.035 |
| | | | **Campylobacter** | 0.397 | 0.740 | 0.443 | 0.605 | 0.562 | 0.086 | 0.583 | 0.564 | 0.835 | 0.672 |
| | | | **Treponema 2** | 0.755 | 0.668 | 0.667 | 0.929 | 0.868 | 0.075 | 0.725 | 0.200 | 0.255 | 0.000 |
